# An Atypical Case of Miller Fisher Syndrome

**DOI:** 10.7759/cureus.74212

**Published:** 2024-11-22

**Authors:** Atteh Akoto, Elizabeth Sain

**Affiliations:** 1 Emergency Medicine, Summa Health, Akron, USA; 2 Internal Medicine, Summa Health, Akron, USA

**Keywords:** emergency medicine, emergency visits, guillan-barre syndrome, lumbar puncture (lp), miller fisher syndrome (mfs), neurology and critical care, weakness

## Abstract

Miller Fisher syndrome (MFS) is a rare variant of Guillain-Barré syndrome (GBS) characterized by a classic triad of external ophthalmoplegia, ataxia, and areflexia, often following a recent infection.

Understanding atypical presentations of MFS is crucial for timely diagnosis and management, as the syndrome may be mistaken for other neurological disorders. This report aims to highlight the clinical journey of the patient, including symptom onset, diagnostic challenges, and therapeutic interventions, with a discussion of the broader implications of such atypical cases in the context of MFS.

In this case, a 28-year-old male presented with binocular diplopia and numbness. The patient had recently returned from Europe, where he experienced a viral diarrheal infection. He presented to the emergency department with diplopia and numbness throughout his entire body and complained of difficulty ambulating because of the loss of sensation in his feet. The examination was significant for binocular diplopia, bilateral disconjugate gaze with esotropia, diminished sensations, and absent deep tendon reflexes in all four extremities, but no ataxia or weakness was elicited. His case was discussed with neurology with concern for GBS or MFS, and he was hesitantly admitted to the floor with a negative inspiratory force (NIF) of -22 (normal < -60) under the resident admitting service. Lumbar puncture (LP) studies including meningitis/encephalitis pathogen panel, venereal disease research laboratory (VDRL), myelin basic protein, anti-GQ1b antibody, and bacterial cultures were initially unremarkable.

Even despite suspicion of the diagnosis, no treatment was started until neurology started IV immunoglobulin (IVIG) the next day when his symptoms worsened with bilateral knee weakness. He received five days of 400 mg/kg of IVIG with an improvement of his distal weakness, numbness, and diplopia, as well as respiratory function with a NIF of -48. With an improved ability to ambulate, he was discharged without new medications with a presumed diagnosis of GBS as the gastrointestinal (GI) polymerase chain reaction (PCR) test showed *Campylobacter jejuni* and *Salmonella*. Lumbar puncture studies were eventually positive for the anti-GQb1 antibody, which is consistent with the Miller Fisher variant of GBS.

Ultimately, this case exemplifies the need for heightened awareness among healthcare professionals regarding the diverse manifestations of the MFS. As our understanding deepens, we can improve diagnostic accuracy and treatment outcomes for patients suffering from this rare and challenging condition.

## Introduction

Guillain-Barré syndrome (GBS) is an autoimmune neurological disorder characterized by ascending paralysis. Miller Fisher syndrome (MFS) is a rare variant of GBS characterized by a classic triad of external ophthalmoplegia, ataxia, and areflexia, often following a recent infection [1]. Worldwide, the incidence is approximately one to two in 1,000,000 with a predominance of the male gender, Asian race with a mean age in the early 40s [2]. While traditionally associated with antecedent illnesses, such as viral gastroenteritis or respiratory tract infections, which are theorized to trigger an autoimmune response, the presentation of MFS can vary significantly among individuals [3]. Typically, MFS starts after a 10-day incubation period and progresses over days to weeks, with the peak of symptoms at around four weeks. Traditionally, all three of the triad manifest at some point during this course, but diplopia, dizziness, paresthesias, distal and cranial nerve weakness, as well as autonomic dysfunction, can also be a part of symptomatology [2]. Diagnosis is confirmed by lumbar puncture (LP), typically by albuminocytologic disassociation, where there is an increase in protein levels in the cerebrospinal fluid (CSF) but a normal number of nucleated cells, but this is not always present, as in this case. Antibody testing, nerve conduction studies, electromyography, and neuroimaging of the spine can also aid in diagnosis. Treatment is by immunoglobulin therapy or plasma exchange, neither of which is inferior to the other. Both work by removing from the blood and body the antibodies attacking the nervous system [2]. This case report details an atypical presentation of MFS in a previously healthy adult who exhibited an unusual progression of symptoms not commonly associated with this condition.

Understanding atypical presentations of MFS is crucial for timely diagnosis and management, as the syndrome may be mistaken for other neurological disorders such as Wernicke encephalopathy, ocular myasthenia gravis, Lambert Eaton syndrome, multiple sclerosis, and sarcoidosis, among other autoimmune disorders, potentially delaying appropriate treatment [2]. Current literature notes concurrent vaccinations, immune thrombocytopenic purpura, and cranial nerve palsies that can result from MFS [4-6]. The literature also notes that there are specific antibodies that can clue into the diagnosis of MFS. Anti-GQ1b antibodies are immunological markers produced in the central and peripheral nervous system in response to microbial infections, such as *Haemophilus influenzae *and *Campylobacter jejuni*. The antibodies bind to GQ1b antigens in the central nerves, resulting in a spectrum of autoimmune diseases typically associated with ophthalmoplegia [3].

This report aims to highlight the clinical journey of the patient, including symptom onset, diagnostic challenges, and therapeutic interventions, with a discussion of the broader implications of such atypical cases in the context of MFS. By presenting this unusual case, we hope to contribute to the existing literature and enhance awareness of the diverse clinical manifestations of MFS among healthcare professionals.

## Case presentation

A 28-year-old male presented with binocular diplopia and numbness. The patient had recently returned from Europe, where he experienced a viral diarrheal infection. Three days prior to arrival, he started having binocular diplopia and complained of decreased sensation in his bilateral hands. He was evaluated at an outside hospital with CT angiography of the head and neck, and laboratory investigations, including a complete blood count and complete metabolic panel, were all unremarkable. He woke up on the morning of his presentation to the emergency department with worsening diplopia and numbness throughout his entire body. He denied weakness in any of the extremities, however, he complained of difficulty ambulating because of the loss of sensation in his feet. The examination was significant for binocular diplopia, bilateral disconjugate gaze with esotropia, diminished sensations, and absent deep tendon reflexes in all four extremities, but no ataxia or weakness was elicited. His case was discussed with neurology due to concern for GBS or MFS, and he was hesitantly admitted to the floor with a negative inspiratory force (NIF) of -22 (normal < -60) under the resident admitting service. An MRI of the brain showed incidental pituitary adenoma without prolactinemia, favored to be a Rathke cleft cyst. Lumbar puncture studies including a meningitis/encephalitis pathogen panel, venereal disease research laboratory (VDRL), myelin basic protein, anti-GQ1b antibody, and bacterial cultures were initially unremarkable (Table 1).

**Table 1:** The patient's cerebrospinal fluid (CSF) studies

CSF cell counts	Value	Reference range
White blood cell (WBC)	2 cells/mm^3^	<5 cells/mm^3^
Red blood cell (RBC)	1 cells/mm^3^	0 cells/mm^3^
Glucose	58 mg/dL	40-70 mg/dL
Protein	31.7 mg/dL	12.0-60.0 mg/dL

Neurology started intravenous immunoglobulin (IVIG) the next day when his symptoms worsened with bilateral knee weakness. He received five days of 400 mg/kg of IVIG with improvement of his distal weakness, numbness, and diplopia, as well as respiratory function with a NIF of -48. With an improved ability to ambulate, he was discharged without new medications with a presumed diagnosis of GBS as GI polymerase chain reaction (PCR) showed *Campylobacter jejuni *and *Salmonella*. Lumbar puncture studies were eventually positive for the anti-GQ1b antibody, which is consistent with the Miller Fisher variant of GBS.

## Discussion

Miller Fisher syndrome is a variant of GBS. Unlike GBS, which usually presents with ascending paralysis, MFS is typically associated with ophthalmoplegia, ataxia, and areflexia rather than limb weakness. However, some cases, like this one, do not present with the full triad but instead, sensory or peripheral muscular strength deficits. Both immune-mediated peripheral nerve demyelinating variants are most often preceded by *Campylobacter *infection; in this case, however, one must have a high index of suspicion as CSF studies can be negative for the typical albuminocytologic disassociation, an increased protein concentration with a normal white blood cell count [7]. Though other similar syndromes such as myasthenia gravis, Lambert Eaton syndrome, and chronic inflammatory demyelinating polyneuropathy also respond to IVIG, diagnosis can be confirmed with anti-GQ1b antibodies, which is specific to only a handful of diagnoses on a spectrum of anti-GQ1b antibody syndrome [8]. These antibodies can also be present in Bickerstaff brainstem encephalitis (BBE) and pharyngeal-cervical-brachial (PCB) wеаknеss. Like MFS, BBE is associated with opthalmoplegia and ataxia but also presents with encephalopathy and facial weakness [9]. Pharyngeal-cervical-brachial weakness also often presents with facial weakness but is characterized by acute weakness of the oropharyngeal, neck, and shoulder muscles with swallowing dysfunction [10].

Negative inspiratory force should be obtained in patients with suspected GBS and MFS, as the paralysis can also affect the diaphragm, resulting in respiratory failure, arguably the most important reason why this disease must be caught early, though albeit, case fatality occurs at a rate of less than 5%. Elective intubation should be considered for patients with NIF greater than -30. Noninvasive ventilation can also potentially be used but can be dangerous as it cannot help the patient protect their airway if the disease continues to progress. A fulminant course (onset to admission less than seven days), bulbar weakness, and neck flexion weakness are also predictive of the need for intubation [11].

Treatment is otherwise with IVIG or plasma exchange over five days or sessions, respectively. With treatment, the vast majority of patients do well and recover their function over several weeks to months with physiotherapy, though symptom recurrence is still possible and occurs at a higher rate in MFS than in GBS [11].

## Conclusions

This case report highlights an atypical presentation of MFS in a previously healthy adult characterized by abnormal symptoms not typically associated with MFS, underscoring the importance of recognizing the variant's clinical complexities. The patient’s initial symptoms, including binocular diplopia and sensory deficits despite normal CSF studies, rather than the typical ataxia, areflexia, and ophthalmoplegia, emphasize the need for a high index of suspicion when diagnosing MFS, particularly in cases with unusual symptom progression and presentations. The eventual positive identification of *Campylobacter jejuni* infection, along with the delayed identification of anti-GQ1b antibodies, supports the known association between gastrointestinal illnesses and the development of MFS. 

Early intervention with IVIG therapy was pivotal in this patient’s recovery, illustrating the necessity of prompt treatment to mitigate the risk of further neurological deterioration. The inclusion of negative inspiratory force assessments in the management of patients presenting with symptoms suggestive of GBS or its variants is critical, as respiratory compromise can be a significant threat. Follow-up with physiotherapy is important to regain the strength lost from the disease, though in MFS, the course is usually more benign. The symptoms can recur, though this is less likely when a microbial trigger can be elicited.

Ultimately, this case exemplifies the need for heightened awareness among healthcare professionals regarding the diverse manifestations of MFS. As our understanding deepens, we can improve diagnostic accuracy and treatment outcomes for patients suffering from this rare and challenging condition. Continued research into the variations of GBS and its underlying mechanisms will further enhance our ability to provide effective care for individuals affected by this disorder.

## References

[1] Ueno T, Kon T, Kurihara AI, Tomiyama M (2017). Unilateral oculomotor nerve palsy following Campylobacter infection: a mild form of Miller Fisher syndrome without ataxia. Intern Med.

[2] Rocha Cabrero F, Morrison EH (2024). Miller Fisher Syndrome. https://www.ncbi.nlm.nih.gov/books/NBK507717/.

[3] Taboada EN, van Belkum A, Yuki N (2007). Comparative genomic analysis of Campylobacter jejuni associated with Guillain-Barré and Miller Fisher syndromes: neuropathogenic and enteritis-associated isolates can share high levels of genomic similarity. BMC Genomics.

[4] Wei CQ, Yu X, Wu YY, Zhao QJ (2024). Miller fisher syndrome with positive anti-GQ1b/GT1a antibodies associated with COVID-19 infection: a case report. World J Clin Cases.

[5] Fynke J, Perez N, Wagner B, Zuflacht J (2024). Concurrent Miller Fisher syndrome and immune thrombocytopenic purpura: a case report and review of the literature. Neurohospitalist.

[6] Chen NH, Bin Zainul Abideen A, Wee TC (2024). Delayed onset bilateral vocal cord palsy in Miller Fisher syndrome: the rehabilitation outcome. Cureus.

[7] Noioso CM, Bevilacqua L, Acerra GM (2023). Miller Fisher syndrome: an updated narrative review. Front Neurol.

[8] Ang CW, De Klerk MA, Endtz HP, Jacobs BC, Laman JD, van der Meché FG, van Doorn PA (2001). Guillain-Barré syndrome- and Miller Fisher syndrome-associated Campylobacter jejuni lipopolysaccharides induce anti-GM1 and anti-GQ1b antibodies in rabbits. Infect Immun.

[9] Odaka M, Yuki N, Yamada M, Koga M, Takemi T, Hirata K, Kuwabara S (2003). Bickerstaff's brainstem encephalitis: clinical features of 62 cases and a subgroup associated with Guillain-Barré syndrome. Brain.

[10] Nagashima T, Koga M, Odaka M, Hirata K, Yuki N (2007). Continuous spectrum of pharyngeal-cervical-brachial variant of Guillain-Barré syndrome. Arch Neurol.

[11] Nguyen TP, Taylor RS (2024). Guillain-Barre Syndrome. StatPearls.

